# A novel carboxyl-terminal protease derived from *Paenibacillus lautus* CHN26 exhibiting high activities at multiple sites of substrates

**DOI:** 10.1186/1472-6750-13-89

**Published:** 2013-10-25

**Authors:** Yunxia Li, Yingjie Pan, Qunxin She, Lanming Chen

**Affiliations:** 1 Key Laboratory of Quality and Safety Risk Assessment for Aquatic Products on Storage and Preservation (Shanghai), China Ministry of Agriculture, Engineering Centre for Quality Control and Risk Assessment of Aquatic Products, College of Food Science and Technology, Shanghai Ocean University, 999 Hu Cheng Huan Road, Shanghai 201306, People's Republic of China; 2 Department of Biology, University of Copenhagen, Ole Maaløes Vej 5, Copenhagen DK-2200N, Denmark

**Keywords:** CtpA, *Paenibacillus*, Protease, Aqueous environment

## Abstract

**Background:**

Carboxyl-terminal protease (CtpA) plays essential functions in posttranslational protein processing in prokaryotic and eukaryotic cells. To date, only a few bacterial *ctpA* genes have been characterized. Here we cloned and characterized a novel CtpA. The encoding gene, *ctpAp* (*ctpA* of *Paenibacillus lautus*), was derived from *P. lautus* CHN26, a Gram-positive bacterium isolated by functional screening. Recombinant protein was obtained from protein over-expression in *Escherichia coli* and the biochemical properties of the enzyme were investigated.

**Results:**

Screening of environmental sediment samples with a skim milk-containing medium led to the isolation of a *P. lautus* CHN26 strain that exhibited a high proteolytic activity. A gene encoding a carboxyl-terminal protease (*ctpAp*) was cloned from the isolate and characterized. The deduced mature protein contains 466 aa with a calculated molecular mass of 51.94 kDa, displaying 29-38% amino acid sequence identity to characterized bacterial CtpA enzymes. CtpAp contains an unusual catalytic dyad (Ser_309_-Lys_334_) and a PDZ substrate-binding motif, characteristic for carboxyl-terminal proteases. CtpAp was expressed as a recombinant protein and characterized. The purified enzyme showed an endopeptidase activity, which effectively cleaved α S1- and β- casein substrates at carboxyl-terminus as well as at multiple internal sites. Furthermore, CtpAp exhibited a high activity at room temperature and strong tolerance to conventional protease inhibitors, demonstrating that CtpAp is a novel endopeptidase.

**Conclusions:**

Our work on CtpA represents the first investigation of a member of Family II CtpA enzymes. The gene was derived from a newly isolated *P. lautus* CHN26 strain exhibiting a high protease activity in the skim milk assay. We have demonstrated that CtpAp is a novel endopeptidase with distinct cleavage specificities, showing a strong potential in biotechnology and industry applications.

## Background

CtpA proteins cleave peptide bonds at the carboxyl-terminus (C-terminus) of polypeptides, and are primarily involved in posttranslational protein processing, maturation or degradation in prokaryotic and eukaryotic cells [1]. These enzymes belong to an unusual type of serine peptidases that carry a catalytic dyad (Ser-Lys) at the active site, instead of the well-known Ser-His-Asp catalytic triad commonly found in most serine proteases [2,3]. The first CtpA was described in *Escherichia coli* albeit under different identities: either as tail-specific protease (Tsp) or processing involving the C-terminal cleavage (Prc). This *E. coli* enzyme is an endopeptidase which cleaves peptide substrates containing apolar residues and a free α–carboxylate at the C-terminus [4-6]. To date, CtpA enzymes have been characterized in some higher plants, algae, and bacteria [1]. In photosynthetic higher plants and cyanobacteria, CtpA is an essential peptidase in photosystem II (PS II) reaction center [7,8], where the enzyme cleaves a precursor form of D1 protein, a key subunit of PS II, at C-terminal extension which consists of typically 9-16 amino acid residues [1]. Non-photosynthetic bacterial CtpA enzymes exhibit a great diversity in sequence, suggesting that they have gained different functions during evolution. The *E. coli* CtpA is a protein with an apparent mass of about 80 kDa in maxicell and *in vitro* systems [4]. The enzyme cleaves a periplasmic penicillin-binding protein 3 in *E. coli* and is involved in thermal and osmotic stresses and pathogenesis [4,9]. In *Borrelia burgdorferi*, CtpA processes outer-membrane proteins P13 and BB0323 [10], whereas the same enzyme functions in bacterial biofilm formation in *Rhizobium leguminosarum*[11], and is involved in cell morphology and intracellular survival in *Burkholderia mallei* and *Brucella suis*[12,13]. Furthermore, biochemical traits of bacterial CtpA have indicated that these enzymes are resistant to a wide variety of conventional protease inhibitors [5,14], which makes them potential good candidate proteases for biotechnology and industry application. Proteases constitute one of the three largest groups of industrial enzymes, accounting for about 60% of the total sales of enzymes worldwide [15], and they have been used in detergent, food, agrochemical and pharmaceutical industries [16]. Microorganisms are the main resources of hydrolases from which most commercial hydrolases are derived [17]. This is mainly because microbes exhibit much greater diversity than multicellular organisms (e.g. [18]).

As aquaculture industry is a fast developing area in China, sediments of aquaculture ponds have developed into novel environmental niches that are very rich in different nutrients, allowing diverse microbes to thrive. Recently a highly active esterase with exceptional resistance to organic solvents has been obtained from a sample collected from the environment [18]. Here we screened for protease-producing bacteria from sediment samples collected from the same fishery ponds in Shanghai, China, and found one of the isolates, *Paenibacillus lautus* CHN26, exhibited a high protease activity. A *ctpA* gene was cloned from this bacterium and expressed in *E. coli* using the pET-28a expression system. The resulting recombinant CtpA protein was purified and characterized. Our data indicated that *P. lautus* CtpA is a novel endopeptidase, exhibiting a strong application potential in biotechnology.

## Results and discussion

### Screening and identification of a protease-producing bacterium

**Figure 1:**
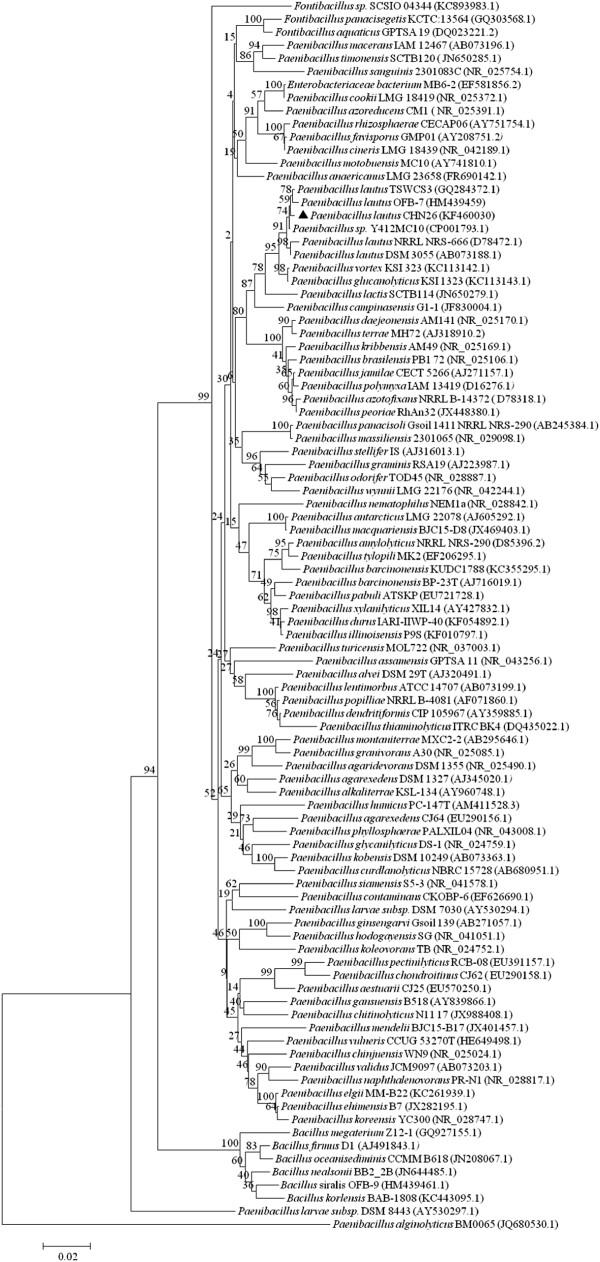
**Phylogenetic anlysis of 16S rRNA gene sequences of all *Paenibacillus *species showing that the newly isolated CHN26 is most closely related to *Paenibacillus lautus*.** Bootstrap percentages are shown at nodes. The scale bar represents 0.02 changes per nucleotide. The GenBank accession numbers are shown behind the names of the strains, and the strain identified in this study is marked with a solid triangle.

We have developed a functional screening assay in which extracellular protease-producing bacteria form clear zones around their colonies on selective skim-milk agar plates (see the Methods). Employing the assay to screen for protease-producing bacteria from a sediment sample of fishery ponds identified several positive colonies, among which the isolate CHN26 produced very large clear zone of hydrolysis on the selective plate, suggesting that it could encode one or multiple highly active protease(s). Its 16S rRNA gene was amplified by polymerase chain reaction (PCR) using the 27 F and 1492R primers, and the gene sequence was determined (GenBank: KF460030). Database searches with the 16S rRNA gene revealed that it displayed 96-99% nucleotide sequence identity with the corresponding genes derived from dozens of *Paenibacillus* bacteria. The phylogenetic position of the isolate was studied by comparing the 16S rRNA gene sequence with a selected set of *Paenibacillus* bacteria retrieved from the GenBank databases and a phylogenetic tree was constructed using the MEGA4.0 (Figure 1). Strain CHN26 fell into the clade of *P. lautus*, suggesting it was closely related to this species.

Bacteria of *Paenibacillus* species are widespread: they have been isolated from different environments including soil, plant, insect and aqueous samples. A very striking feature for these bacteria is that they exhibit great versatility in metabolic capacities. These bacteria produce various extracellular enzymes such as polysaccharide-degrading enzymes and proteases, several of which have shown strong potential in industrial applications [19-23].

Characterization of the *P. lautus* CHN26 revealed the following: Cells of this organism are Gram-positive rods, oxidase- and catalase-positive, and form transparent non-pigmented colonies on nutrient agar media. It grows optimally at 30°C, pH 7.5 and in the presence of 3% NaCl. This strain was negative in the Indole test, and did not produce H_2_S. Neither did it hydrolyze or utilize starch, dextrin, _D_-xylose, _D_-galactose, _D_-ribose, inositol, maleic acid or sodium succinate. The bacterium was tested positive in the Voges-Proskauer, and methyl-red assays. More importantly, it could grow in the medium containing α /β casein, sucrose, _D_-frucose, lactose, _D_-sorbito or glycerol as the sole energy (Table 1). In summary, this isolate showed a few distinct features such as utilization of _D_-sorbito and citrate, Voges-Proskauer test, H_2_S production and nitrite reduction, which deviated itself from the other known *P. lautus* strain [24].

**Table 1:** **Phenotypic features of the *P. lautus *** **CHN26**

**Features**	***Paenibacillus lautus *** **CHN26**^**a**^
Gram-staining	Positive
Cell size (μm)	2-5 mm
Colony colour and morphology	No pigment, circular, smooth, slightly convex, margin entire,
Temperature for growth (°C)	15-40, optimum: 30
pH for growth	5.5-8.5, optimum: 7.5
NaCl concentration for growth (%, w/v)	1-5, optimum: 3
Hydrolysis of starch	-
Assimilation of: _L_-arabinose, Citrate, _D_-glucose, maltose	+
Utilization of:	
Α/β casein	+
Sucrose, _D_-frucose, lactose, _D_-sorbito	+
_D_-xylose, _D_-ribose, _D_-galactose,	-
Sodium succinate, inositol, maleic acid	-
Glycerol	+
Voges-Proskauer test, methyl-red test, H_2_S production, oxidase, gelatin liquefaction, nitrite reduction, phenylalanine deaminase,	+
Indole test, producion of dextrin	-

^**a**^+ : positive reaction; -:negative reaction.

### Molecular cloning of a *ctpA* gene in *Paenibacillus lautus* CHN26

We were interested in studying proteases encoded in this bacterium. Recently, the complete genome of *Paenibacillus sp.*Y412MC10 was reported (GenBank: NC_013406.1) [25]. To gain an insight into what gene product could be responsible for the hydrolytic activity of *P. lautus* CHN26 in the skim milk assay, a few putative protease genes of *Paenibacillus* were investigated, in which the C-terminal protease could be responsible for the observed activity (see below).

*P. lautus* encodes only one putative C-terminal protease, although many other *Bacillus* species (e.g. *B. subtilis*) encodes two, namely CtpA and CtpB. A few CtpB enzymes have been characterized and it has been shown that these enzymes play important functions in cellular process such as sporulation [26]. However, to our knowledge, none of CtpA enzymes derived from a Gram-positive bacterium have been characterized thus far.

**Figure 2:**
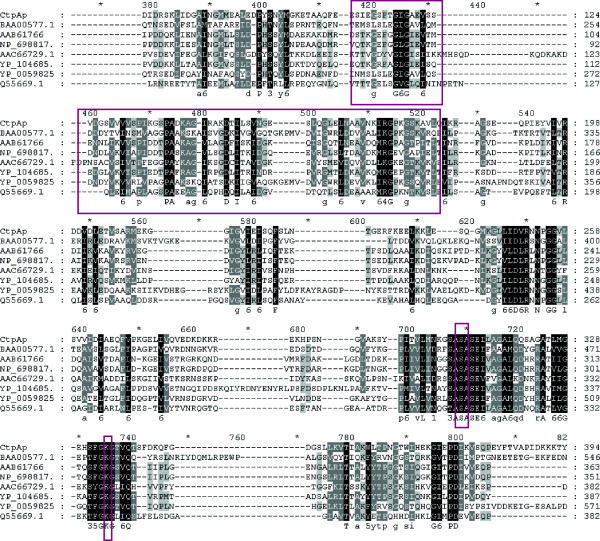
**A multiple sequence alignment of *P. lautus *CtpA and a selected set of bacterial CtpA proteins.** Numbers above the alignments indicate relative positions of the entirely aligned sequences. Identical amino acid residues as well as the conserved ones (>50% of the sequences) are highlighted in black and in grey, respectively, with the consensus sequence shown below the alignment. The catalytic dyad and PDZ motif are boxed. The CtpA sequences chosen for the analysis are obtained from GenBank and NCBI databases: BAA00577.1: *E. coli* K-12; AAB61766: *B. bacilliformis* KC583; NP_698817.1: *B. suis* 1330; AAC66729.1: *B. burgdorferi* B31; YP_104685.1: *B. mallei* ATCC23344; YP_005982591.1: *P. aeruginosa* NCGM2.S1; Q55669.1: *Synechocystis* sp. PCC 6803 substr. GT-1; CtpAp (GenBank: KF169841): *P. lautus* CHN26, obtained in this study.

The *ctpA* gene from *P. lautus* CHN26 was amplified using the primer pair ctp1-F and ctp1-R (see the Methods) targeting the corresponding gene sequence of *Paenibacillus sp.*Y412MC10 (GenBank: NC_013406.1), which was re-classed as *P. lautus* recently [24,25]. A PCR product of the expected size (1.4 kb) was obtained from *P. lautus* CHN26, and the *ctpA* gene sequence of *P. lautus* CHN26 was determined (GenBank: KF169841): an open reading frame of 1,473 bp encoding a protein of 490 aa with a calculated molecular mass of 53.92 KDa. The deduced amino acid sequence displayed high sequence identities to putative C-terminal proteases encoded in *Paenibacillus* species (53-99% identity), all of which have not been characterized. Furthermore, *P. lautus* CtpA showed relatively lower sequence similarities (29-38% identities) to all characterized bacterial CtpA proteins, including those encoded in *E. coli*, *Bartonella bacilliformis*, *B. burgdorferi, B. suis*, *B. mallei*, and *Pseudomonas aeruginosa*[4,12,13,27-29]. These CtpA sequences were retrieved from the GenBank database and analyzed by multiple sequence alignments. This analysis revealed that CtpA of the *P. lautus* (CtpAp) contains the catalytic dyad (Ser_309_-Lys_334_) and substrate-binding B domain, also designated as PDZ motif [2,3], both of which are highly conserved in all known CtpA proteins (Figure 2).

**Figure 3:**
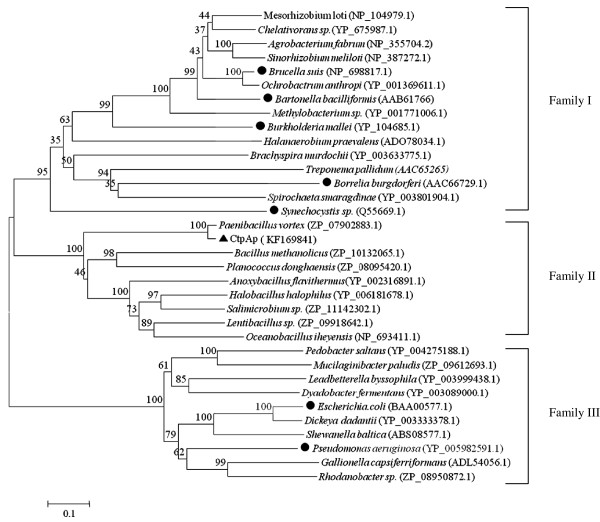
**Phylogenetic tree showing evolutionary relationship between the *P. lautus *CtpA and all characterized bacterial CtpA and related proteins.** A neighbor-joining phylogenetic tree was constructed using MEGA 4.0. Bootstrap percentages are shown at nodes. The scale bar represents 0.1 changes per amino acid. Filled cycles denote the bacterial CtpA proteins that have been characterized for their activity and function, whereas *P. lautus* CtpA identified in this study is marked with a solid triangle.

The multiple sequence alignments were also used to construct phylogenetic trees to gain an insight to their diversity and classification. This analysis revealed three families for CtpA enzymes, namely Family I, II, and III (Figure 3). The CtpA enzymes of *E. coli* and *P. aeruginosa* belong to family I, the enzymes encoded in *B. bacilliformis*, *B. burgdorferi*, *B. suis*, *B. mallei* and *Synechocystis* sp. fall into Family III, whereas CtpAp forms family II with several CtpA entries identified in the GenBank databases. Notably, family II of CtpA proteins are derived from diverse bacterial species phylogenetically distantly related.

### Expression and purification of the CtpAp protein in *E. coli* BL21

**Figure 4:**
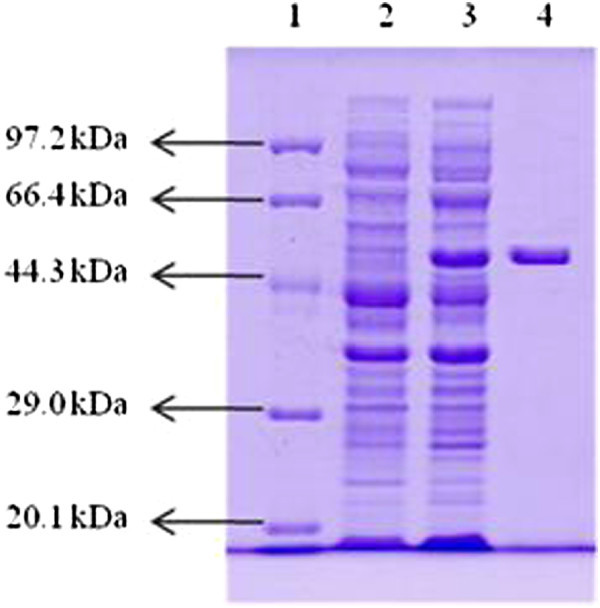
**SDS-PAGE analysis of the *P. lautus ctpA *gene product.** Protein molecular weight marker (Tiangen, Cat.No. DPP530S) (Lane 1), soluble proteins of *E. coli* BL21 (pET28a) (Lane 2) and *E. coli* BL21 (pET28a-*ctpAp*) (Lane 3) cultured and induced (ITPG, 1 mM) under the same condition at 20°C for 18 h, and purified recombinant *P. lautus* CtpA protein (Lane 4).

The *ctpAp* structural gene (1398-bp, excluding the first 72-bp signal peptide sequence of the 5’-terminus and the stop codon) was amplified with the primer pair ctp1-F2 and ctp1-R2 (see the Methods). The PCR product was cloned into the expression vector pET-28a, giving pET28a-*ctpAp*. The expression plasmid was transformed into *E. coli* BL21, and Kan^r^ transformants were screened by colony PCR to test for the presence of pET28a-*ctpAp*. Positive transformants were cultured in LB-kanamycin medium, and the expression of *ctpAp* gene was analyzed by SDS-PAGE. As presented in Figure 4 (lane 3), one band appeared at the size corresponding to the calculated molecular mass of CtpAp in the pET28a-*ctpAp* transformant, but absent from the *E. coli* BL21 cells carrying pET28a (Figure 4, lane 2), suggesting that the protein band represented the recombinant CtpAp resulting from episomic *ctpAp* expression.

The recombinant CtpAp protein with twelve his-tags was purified by affinity purification via a Ni-NTA His Bind resin column. Then, the purified enzyme was concentrated and desalted using an Amicon Ultra-15 centrifugal filter device. Figure 4 (lane 4) shows the purified CtpAp protein with an apparent molecular mass of approximately 52 KDa, giving a protein yield of about 50.64 mg of per 1 g wet biomass and a relative activity of 764.78 U/mg protein using β-casein as the substrate.

### Substrate specificity of the *P. lautus* CtpA enzyme

To examine protease activity of the recombinant CtpAp protein, *E. coli* BL21 strain harboring the expression plasmid pET28a-*ctpAp* was streaked on the skim milk agar plates containing 30 μg/ml kanamycin and IPTG, and incubated at 37°C for 36 h. Clear zones of hydrolysis around the pET28a-*ctpAp* transformant appeared on the selective agar plates. The colonies of *E. coli* BL21 containing only pET28a did not form any clear zones in the skim milk assay (data not shown).

**Figure 5:**
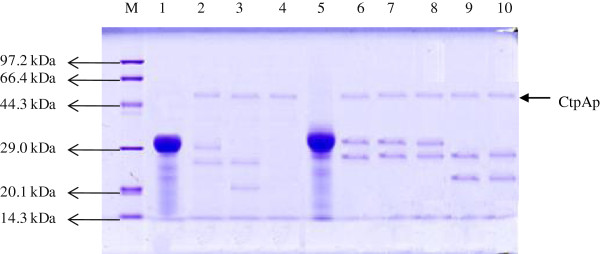
**SDS-PAGE analysis of degradation of the casein substrates by *P. lautus *CtpA.** Protein molecular weight marker (Tiangen, Cat.No. DPP530S) (Lane M); The α S1-casein (Lane 1) was incubated with CtpAp at 30°C for 0.5 (Lane 2), 1 (Lane 3) and 1.5 h (Lane 54), respectively; while the β-casein (Lane 5) with the enzyme at 30°C for 0.5 (Lane 6), 1 (Lane 7), 2 (Lane 8), 4 (Lane 9) and 6 h (Lane 10), respectively.

The purified CtpAp enzyme was then assayed for proteolytic activity using two different casein substrates, β-casein and α S1-casein. The purified enzyme was incubated with each substrate at 30°C for 0.5-6 h, and reaction products were analyzed by SDS-PAGE. As shown in Figure 5, CtpAp cleaved β-casein into shorter fragments (Figure 5, Lane 6-10) as for other bacterial CtpA enzymes (e.g. Spiers et al., 2002). Notably, α S1-casein appeared to serve as a better substrate for CtpAp degradation than β-casein, as the reaction with the former substrate was ca. 3-fold faster than the degradation of the latter substrate under the same reaction condition (Figure 5, Lane 2-4). This result is in a good agreement with the high protease activity of *P. lautus* CHN26 in the skim milk assay since α S1-casein is a predominant component of the substrates in the assay.

To reveal the cleavage sites of CtpAp on the casein substrates, we analyzed the digest products using a matrix-assisted laser desorption and ionization time of flight mass spectrometer (MALDI-TOF MS) with the resulting data summarized in Table 2. CtpAp cleavage of both substrates yielded multiple peptide fragments of different sizes, suggesting that cleavage by CtpAp lacked stringency in site selection on each substrate. CtpAp cleaved β-casein (224 aa residues, Swiss-Prot: P02666.2) [30] at four sites at the C-terminus, including Glu_210_-Pro_211_, Leu_213_-Gly_214_, Gly_214_-Pro_215_ and Pro_215_-Val_216_, all of which are apolar amino acid residues. It has been reasoned that CtpA enzymes rely on the recognition of scissile bonds at C-terminus of the substrates for C-terminal protease activity, giving typically oligopeptide products of 9-14 residues. Such cleavage preferences have been reported previously for *E. coli* CtpA enzyme [4-6]. Similarly, for the α S1-casein substrate of 214 aa (GenBank: 1308122A) [31], the enzyme recognized three peptide bonds at C-terminus and produced oligopeptides of 7-9 aa. However, interesting differences in site selection on α S1-casein were observed: all three cleavage sites of CtpAp contain polar residues at C-terminus of the substrate (Asn_205_-Ser_206_, Ser_206_-Glu_207_ and Glu_207_-Lys_208_), namely Asn, Ser and Lys, which is in contrast to its site selection on β-casein where only nonpolar residues are sites of cleavage (see above).

**Table 2:** **Cleavage of β and α S1-casein substrates by the *P. lautus *** **CtpA determined by MALDI-TOF MS**

**Substrates**	**Monoisotopic mass of neutral peptide Mr (cal)**	**Sequences**	**Cleavage sites**	**Expect value**
β-casein	1624.8308	A↓RELEELNVPGEIVE	Ala_15_-Arg_16_	0.000038
	1528.7926	Q↓TQSLVYPFPGPIPN	Gln_69_-Thr_70_	0.0011
	1299.6863	Q↓SLVYPFPGPIPN	Gln_71_-Ser_72_	0.00003
	876.4739	V↓MGVSKVKE	Val_107_-Met_108_	0.027
	947.5287	Q↓SVLSLSQSK	Gln_175_-Ser_176_	0.0015
	994.6175	Q↓SKVLPVPQK	Gln_182_-Ser_183_	0.0027
	1459.8915	E↓PVLGPVRGPFPIIV	Glu_210_-Pro_211_	0.0037
	1150.6863	L↓GPVRGPFPIIV	Leu_213_-Gly_214_	0.0022
	1093.6648	G↓PVRGPFPIIV	Gly_214_-Pro_215_	0.00011
	996.6120	P↓VRGPFPIIV	Pro_215_-Val_216_	0.0061
α S1-casein	1467.7915	L↓LRFFVAPFPEVF	Leu_35_-Leu_36_	0.016
	1051.5379	F↓FVAPFPEVF	Phe_38_-Phe_39_	0.0011
	760.3967	N↓ELSKDIG	Asn_53_-Glu_54_	0.0041
	753.3769	S↓AEERLH	Ser_130_-Ala_131_	0.00092
	1157.5499	A↓EERLHSMKE	Ala_131_-Glu_132_	0.000021
	1107.5270	N↓SEKTTMPLW	Asn_205_-Ser_206_	0.00045
	1004.5001	S↓EKTTMPLW	Ser_206_-Glu_207_	0.00015
	875.4575	E↓KTTMPLW	Glu_207-_Lys_208_	0.0026

In addition, CtpAp also cleaved at six sites on β-casein located more distantly from the C-terminus, including Ala_15_-Arg_16_, Gln_69_-Thr_70_, Val_107_-Met_108_, Gln_175_-Ser_176_, Gln_175_-Ser_176_ and Gln_182_-Ser_183_. Such internal cleavage also occurred for the α S1-casein substrate where five active sites were revealed for CtpAp cleavage, including Leu_35_-Leu_36_, Phe_38_-Phe_39_, Asn_53_-Glu_54_, Ser_130_-Ala_131_ and Ala_131_-Glu_132_. To our knowledge, among all CtpA enzymes characterized to date, the *E. coli* CtpA represents the only one that also cleaves its substrate at multiple sites [6]. Furthermore, the two CtpA enzymes show a great diversity in internal cleavage sites: the only site that is in common for them is Ala_15_-Arg_16_. Such functional diversity of CtpA enzymes is often advantageous for their application in biotechnology.

**Figure 6:**
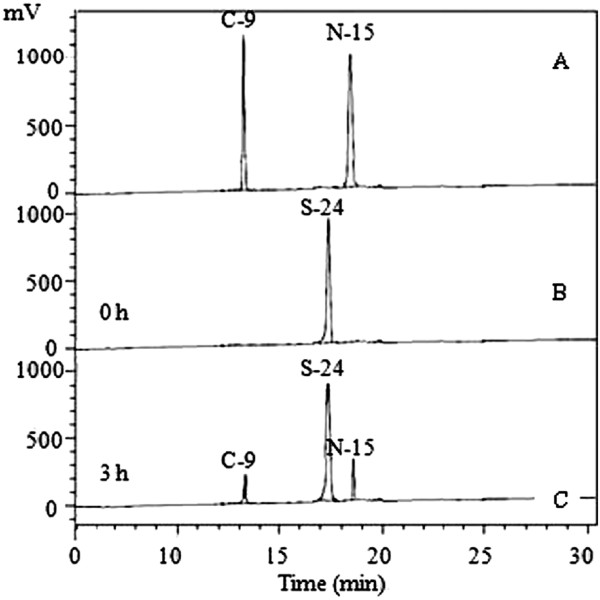
**HPLC analysis of specific cleavage of the C-terminal oligopeptide of pD1 by *P. lautus *CtpA.** The S24 substrate **(B)** corresponding to the 24 aa residues at the C-terminus of pD1 was incubated with CtpAp at 30°C for 3 h, yielding two products N-15 and C-9 oligopeptides **(C)** matching the 15 and 9 residues at the N and C-terminus of the S24 **(A)**, respectively.

In contrast, several known CtpA enzymes have showed strong stringencies in site selection of peptide cleavage. For example, the amino acid residues (Ala-Ala / Ser) at the cleavage sites of C-terminus extension in pD1 are very well conserved in photosynthetic organisms [1,32]. We also tested CtpAp cleavage on this substrate using a synthetic oligopeptide (S24) comprising 24 aa (Val_330_ to Gly_353_) of the pD1 C-terminus. Incubation of the S24 substrate with CtpAp at 30°C for 3 h revealed that this bacterial CtpA enzyme partially broke down the substrate, yielding N15 and C9 oligopeptides (Figure 6C). However, CtpAp appears to be much less active on S24 than that on the casein substrates. In addition, CtpAp failed to show any detectable haemolytic activity. Taken together, CtpA enzymes fall into two distinct categories, in regard of site selection stringency. Photosynthetic CtpA enzymes exhibit strong amino acid sequence specificity and play important cellular functions, whereas nonphotosynthetic CtpAs, such as the *E. coli* CtpA and CtpAp do not show a strong site preference in cleavage, and these enzymes degrade their substrates at multiple sites. Apparently, CtpA enzymes of the latter category show a strong application potential in protein degradation at an industrial level.

### Effect of temperature and pH on *P. lautus* CtpA activity and stability

**Figure 7:**
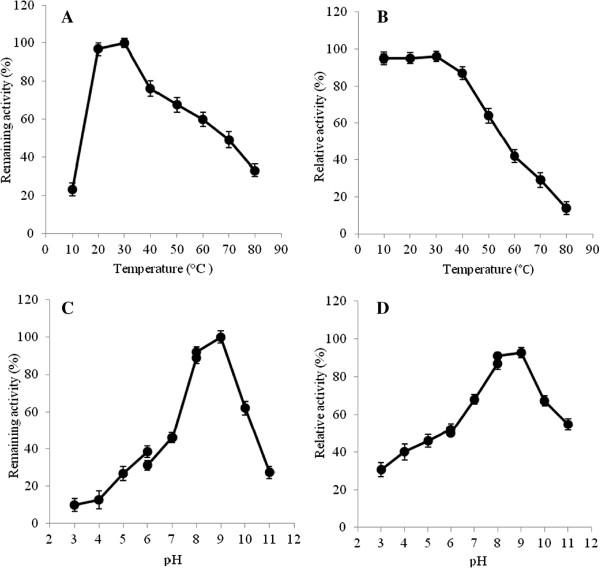
**Effect of temperature and pH on *P. lautus *CtpA enzyme activity and stability.** Relative activity of purified CptAp was determined at different temperatures **(A)** or pH **(C)** using β-casein as the substrate at 562 nm. Remaining enzyme activity was measured at 30°C and pH 9.0 after incubating purified CtpAp at different temperatures for 3 h **(B)**, or pH at 4°C for 12 h **(D)**. All determinants were performed in triplicate.

The temperature profile of CtpAp was determined for the temperature range of 10°C to 80°C at pH 9.0 using β-casein as a substrate. As illustrated in Figure 7A, CtpAp displayed maximum enzyme activity at 30°C, retaining up to 95.6% of its initial activity at 20-35°C. The enzyme activity decreased steeply as the temperature increased above 50°C, indicating that the CtpAp is a mesophilic enzyme.

To examine the effect of temperature on CtpAp stability, aliquotes of the purified enzyme were incubated at various temperatures for 3 h, and the residual activity of the treated protein was assayed at 30°C, pH 9.0. As shown in Figure 7B, CtpAp exhibited considerable stability and retained more than 95% of its initial activity after treatment at 10-30°C for 3 h. Treating the enzyme at 50°C resulted in residual activity of 58%, and further elevating pretreated temperature (60-80°C) yielded a steep reduction of the enzyme activity, suggesting that CtpA is a mesophilic enzyme.

The effect of pH on CtpAp activity and stability was studied at 30°C, the optimal reaction temperature. As shown in Figure 7C, the enzyme displays a high activity at pH 8.0-9.0. The residual activity declined sharply at either higher pH or lower pH values. The similar pH dependency of enzyme activity has been reported for other recombinant CtpA enzymes [1,33]. The effect of pH on enzyme stability was assayed by pre-incubating the purified CtpAp at various pH values for 12 h at 4°C. As shown in Figure 7D, CtpAp exhibited considerable stability at pH 8.0-9.0. However, the enzyme activity diminished more than 50% after prolonged incubation in either more acidic (< pH6.0) or more alkaline (> pH11.0) conditions.

To date, very few previous researches have reported activity of proteases at room temperature [34]. The high activity at room temperature and stability at an alkaline pH render CtpAp as an ideal enzyme in a range of industry application.

### Effect of metal ions and protease inhibitors on *P. lautus* CtpA activity

**Figure 8:**
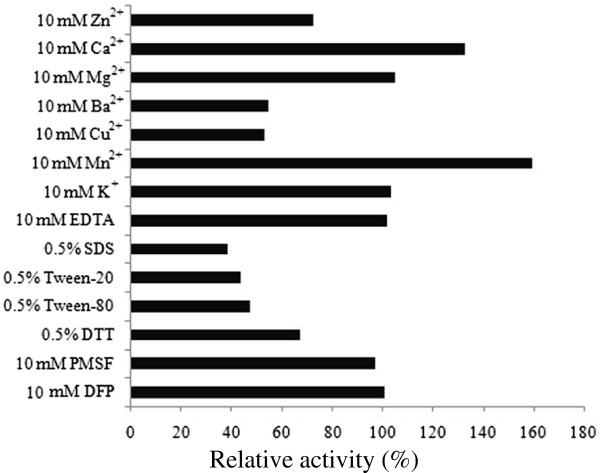
**Effect of metal ions, detergents and inhibitors on *P. lautus *CtpA enzyme stability.** Aliquots of the enzyme were incubated with each metal ion (10 mM), detergent (0.5%) and inhibitors (10 mM) at 30°C for 1 h, the residual activity of CtpAp was determined using β-casein as the substrate. All determinants were performed in triplicate.

To address the effect of metal ions on CtpA activity, aliquotes of the purified enzyme were individually incubated with ZnCl_2_, CaCl_2_, MgCl_2_, BaCl_2_, CuCl_2_, MnCl_2_ and KCl at 1-10 mM concentration at 30°C for 1 h, and residual activities were measured under the standard assay condition (Figure 8). Comparing to the control, Mn^2+^ and Ca^2+^ elevated CtpA activity by 59.6% and 32.8%, respectively, whereas Ba^2+^, Cu^2+^ and Zn^2+^ strongly inhibited enzyme activity, reducing the activity by 30-50%. These results resemble those reported for *E. coli* CtpA [5].

Tolerance to metal chelator (EDTA) and protease inhibitors is very important for any enzymes to be explored in industrial application. As shown in Figure 8, CtpAp displayed remarkable resistance to the conventional inhibitors of serine proteases, as no loss of activity was observed after treating the enzyme with phenylmethanesulfonyl fluoride (PMSF, 1-10 mM) or diisopropyl fluorophosphate (DFP, 1-10 mM), indicating that the enzyme belongs to an unusual type of serine proteases. Protease inhibitor tolerance has been reported for *E. coli* CtpA [5]. However, CtpAp showed much stronger tolerance to the inhibitors tested, since it retained the maximal activity at inhibitor concentrations that were 5-20-fold higher than those reported for *E. coli* enzyme, further arguing for the diversity in biochemical properties of CtpA enzymes. In addition, treating the enzyme with 10 mM EDTA did not yield any effect on the enzyme activity, suggesting that CtpAp is not a metalloprotease.

### Effect of detergents and organic solvents on *P. lautus* CtpA activity

In contrast to distinct stability of CtpAp in the presence of protease inhibitors, detergents including sodium dodecyl sulfate (SDS), Tween 20 and Tween 80 greatly inhibited the enzyme activity, reducing about 53-62% of its activity at a final concentration of 0.5% (Figure 8). Likewise, CtpAp was also sensitive to all the organic solvents tested in this study including acetone, acetonitrile, 1-butanol, ethanol, methanol, 2-propanol, dimethyl sulfoxide (DMSO) and dimethylformamide (DMF), as it lost 42-72% activity under the treatments with each of the organic solvents at a final concentration of 1% (data not shown).

## Conclusions

In this study, a novel carboxyl-terminal protease gene was identified from a newly isolated *Paenibacillus lautus* CHN26 strain, designated *ctpAp*. This gene encodes a mature protein of 466 aa with a calculated molecular mass of 51.94 kDa, displaying 29-38% amino acid sequence identity to a few characterized bacterial CtpA enzymes. CtpAp was expressed as a recombinant protein in *Escherichia coli* BL21 using the pET expression system. The purified CtpAp protein displayed endopeptidase activity, and it effectively cleaves β- and α S1- casein substrates at either C-terminal or multiple internal sites. Comparing CtpAp with other characterized CtpA proteins revealed interesting diversity in their cleavage site selection, and CtpAp also exhibited high activity at a room temperature with strong tolerance to protease inhibitors. The distinct biochemical properties positioned CtpAp as a novel endopeptidase with an application potential in industry and biotechnological applications.

## Methods

### Bacterial strains, plasmids, media and culture conditions

*Escherichia coli* TOP10 [genotype: F^-^*mcr*A∆ (*mrr*-*hsd*RMS-*mcr*BC) ψ80 *lac*Z∆M15∆*lac*X74 *rec*A1 *ara*D139∆ (*ara-leu*) 7697 *gal*U *gal*K *rps*L(Str^r^) *end*A1 *nup*G] (TianGen Biotech Co. Ltd. Beijing, China) was used as a host strain for DNA cloning. *E. coli* BL21 (DE3) [genotype: F^-^*omp*T *hsd* S_B_(*r*_B_^-^*m*_B_^-^) *gal dcm*(DE3) ] (Tiangen) was employed as a host for expression of recombinant proteins. The plasmid pGM-T (TianGen) and pET-28a (Merck Millipore, Darmstadt, Germany) were used as vectors for TA cloning and recombinant protein expression, respectively. *E. coli* was routinely grown in Luria-Bertani (LB) medium [35] at 37°C with shaking at 200 rpm, while *E. coli* strains containing recombinant plasmids were cultured in LB broth or agar plates supplemented with ampicillin (100 μg/ml) or kanamycin (30 μg/ml).

The skim milk agar medium [36] containing 2% (w/v) skim milk (Baomanbio Co. Ltd, Shanghai, China) was used in the skim milk assay to screen for protease-producing isolates. Blood Agar Plates containing 8% (v/v) Defiber sheep blood (Truelab Biotech Co. Ltd, Shanghai, China) was used for haemolysis assay.

### Isolation and characterization of a protease-producing bacterium

Sediment samples were collected from a few aquaculture ponds of fishery farms located in Shanghai, China. Samples were homogenized in nine volumes of a sterilized PBS solution buffer (pH 7.4) [35], and microbial cells in supernatant were collected as previously described by Zhang et al. [37]. Serial dilutions were made and spread onto the selective skim milk agar plates, which were incubated at 30°C for 48 h. Single colonies were scored positive for protease activity using a skim milk assay in which the isolates form clear zone around their colonies resulting from skim milk hydrolysis are scored as positive in protease activity. Conventional phenotypic and biochemical characterizations were carried out for new isolates according to the Bergey’s manual of determinative bacteriology [38] using the microscope (model 36XV, Shanghai Wanheng Precision Instrument Co. Ltd, Shanghai, China) and the bacterial biochemical test kits (Hangzhou Tianhe Microorganism Reagent Co. Ltd., Hangzhou, China). The 16S rRNA gene from the isolate was amplified and sequenced using the bacterial universal primers 27 F (5’-AGAGTTTGATCCTGGCTCAG-3’) and 1492R (5’-TACCTTGTTACGACTT-3’) [39].

Automated DNA sequencing was carried out using ABI3730XL sequencer (Applied Bio-systems, USA) and BigDye Terminator version 3.1 kit at the China Human Genome Center (Shanghai, China). The sequences were analyzed by the program Bioedit (Version 7.0.9, http://www.mbio.ncsu.edu/BioEdit) and the Basic Local Alignment Search Tool (BLAST) (http://www.ncbi.nlm.nih.gov/BLAST). Multiple sequence alignments were performed using the ClustalW2 software (http://www.ebi.ac.uk/Tools/msa/clustalw2/) [40]. The neighbor-joining method in the molecular evolutionary genetic analysis software package MEGA (version 4.0) [41] was used to construct a phylogenetic tree. A bootstrap analysis with 1000 replicates was carried out to check the reliability of the tree. Signal peptide sequences were identified using the SignalP-NN Version 4.0 Server [42]. Oligonucleotide primers were synthesized by Shanghai Sangon Biological Engineering Technology Services Co., Ltd. (Shanghai, China).

### PCR amplification of the *CtpA* gene of the *P. lautus* CHN26

Chromosomal DNA was prepared using the MiniBEST Bacteria DNA Extraction Kit Version 2.0. (Japan TaKaRa BIO, Dalian Company, China) according to the protocols described by the manufacture. The concentration of DNA in the samples was determined using a multi-mode microplate reader BioTek Synergy™ 2 (BioTek Instruments, Inc., VT, USA). The *ctpAp* gene of *P. lautus* CHN26 was amplified by PCR with the primer pair ctp1-F (5’-TTGCTGAAAAAACGAACGG-3’) and ctp1-R (5’-TTACTTGTTCGACGCCTTAGC-3’), which was designed using the software Primer 5.0 (http://www.PremierBiosoft.com) based on the putative C-terminal protease gene sequence of the *Paenibacillus* sp. Y412MC10 (GenBank: NC_013406.1) in the public database. The PCR amplification was performed according to the method described by Shi et al. [18], except that the primer annealing was set at 59°C for 30 s, and elongation at 72°C for 90 s. A sample (5 μl) of each PCR product was analyzed by agarose gel electrophoresis with a 1.0% agarose gel. Amplified DNA fragments were visualized under short-wave UV light (260 nm) and imaged by UVEC_3_ Imaging System (UVP LLC, CA, USA). The PCR product was purified using AxyPrep DNA Gel Extraction Kit (Axygen, CA, USA), and ligated into the pGM-T vector according to the method described by Shi et al. (2013). A 100 μl of transformation culture was spread onto LB-ampicillin plates containing 40 μl 5-bromo-4-chloro-3-indolyl-β-D-galactopyranoside (X-gal, 20 mg/ml), and 16 μl isopropyl-β-D-thiogalactoside (IPTG, 50 mg/ml). Plates were incubated overnight at 37°C, and white colonies were randomly picked up for colony PCR analysis. Plasmid DNA was prepared using the MiniBEST Plasmid DNA Extraction Kit Ver.2.0 (TaKaRa).

### Expression and purification of CtpAp in *E. coli* BL21

Based on the sequence obtained in this study, the primers ctp1-F2 (5’-CC*GGAATT*CATGTTGACCCAGC-3’) and ctp1-R2 (5’-CC*GCTCGA*GCTTGTTCGACG-3’) targeting *ctpAp* structural gene were designed, in which the recognition sites of restriction endonuclease *Eco*RI and *Xho*I were marked as italics. The amplified PCR product and the pET-28a plasmid DNA were individually digested with *EcoR*I and *Xho*I (TaKaRa), purified, and ligated as previously described by Shi et al. [18]. Ligation DNA was transformed into *E. coli* BL21 competent cells via the heat-shock method. A 100 μl of transformation culture was spread onto each LB agar plate containing 30 μg/ml kanamycin. Plates were incubated overnight at 37°C, and colony PCR was used for screening positive clones.

The recombinant CtpAp protein was induced and purified as previously described by Shi et al. [18]. The cell lysate was centrifuged at 16,000 g for 20 min at 4°C, and the supernatant containing soluble target protein was collected and analyzed by one-dimensional sodium dodecyl sulfate-polyacrylamide gel electrophoresis (SDS-PAGE) with 12% separation gel and 5% stacking gel using a Mini-PROTEAN® electrophoresis cell (Bio-Rad). Following electrophoresis, the gel was stained with 0.25% Coomassie brilliant blue R250 and then destained according to the standard method (Sambrook and Russell, 2001). The cell-free extract was purified using Ni-NTA His · Bind® resin (Merck Millipore). The resulting protein fractions were collected and analyzed by SDS-PAGE. The purified target protein was concentrated and desalted using the Amicon Ultra-15 centrifugal filter device (EMD Millipore Corporation, MA, USA) according to the instruction of the manufacturer. The *E. coli* BL21 carrying the vector pET28a was used as a control.

### Protease assays

Cleavage of protein substrates by CtpAp was assayed according to the method described by Spiers et al. [43]. Briefly, 10 μl of purified enzyme and 6 μg casein substrates (β-casein from bovine milk, Sigma-Aldrich, MO, USA; α S1-casein from bovine milk [≥80%], Sangon) in 100 μl of reaction buffer (0.05 M Tris-HCl, 0.5 mM MgCl_2_, pH 9.0) were incubated at 30°C for 0.5-6 h. The reaction products were analyzed by SDS-PAGE. Protease activity was quantitatively measured spectrophotometrically according to the method described by Kasana and Yadav [44]. Briefly, 10 μl of purified enzyme was added into 140 μl reaction buffer (0.05 M Tris-HCl, 0.5 mM MgCl_2_, pH 9.0) containing 4 μl β-casein. After incubation at 30°C for 2 h, the enzyme reaction was terminated by adding 150 μl 10% trichloroacetic acid (TCA). The reaction mixture was centrifuged at 16,000 g for 20 min at 4°C, and 20 μl of supernatant was measured at 562 nm using a microplate reader Synergy™ 2 (BioTek). One relative activity unit was defined as a 0.01 increase in the A_562_ after 2 h at 30°C and pH 9.0 (Kim and Lei, 2005).

The cleavage of peptides was examined by reverse-phase High Performance Liquid Chromatography (HPLC) (model HPX-87P, Bio-Rad, CA, USA). Three oligopeptides were tested for CtpAp cleavage including S-24 (pD1) (Val-Met-His-Glu-Arg-Asn-Ala-His-Asn-Phe-Pro-Leu-Asp-Leu-Ala-Ala-Ile-Glu-Ala-Pro-Ser-Thr-Asn-Gly) corresponding to the C-terminal 24 aa of spinach pD1, as well as N15 (Val-Met-His-Glu-Arg-Asn-Ala-His-Asn-Phe-Pro-Leu-Asp-Leu-Ala) and C9 (Ala-Ile-Glu-Ala-Pro-Ser-Thr-Asn-Gly) [45]. These oligopeptides were synthesized in Sangon (Shanghai, China). The assay was performed according to the methods described by Yamamoto et al. [14]. Briefly, 6 μl of purified CtpAp was added into 33 μl reaction buffer (25 mM Hepes-KOH buffer, pH 7.7) containing 300 μM synthetic peptide substrates, and incubated at 30°C for 3 h. The reaction was terminated by addition of 6 μl of 18% (w/v) TCA, and then the mixture was kept at the room temperature for 20 min. The yielded products were centrifuged at 20,000 *g* for 20 min, 35 μl of the supernatant was mixed with 145 μl of 0.1% (v/v) trifluoroacetic acid (TFA), and the mixture was filtered through the 0.22 μm Millex-GP (Merck Millipore Corporation, Billerica, MA, USA). The 40 μl of the filtrate was analyzed by HPLC analysis with a Vydac 218TP™ C_18_ reversed-phase column (4.6 mm i.d. × 25 cm, 5 μm particle size with 300 Å pore diameter silica) with a linear gradient of 0.2-80% acetonitrile with 0.1% TFA (w/v) at a flow rate of 1 ml/min over 25 min. The peptide peaks were detected at a wavelength of 214 nm in a Photo-Diode Array detector.

Haemolysis assay was performed according to the method described by Mitchell and Minnick [27] by streaking *E. coli* BL21 containing pET-28a-*ctpAp* onto blood agar plates. After incubation overnight at 37°C, the plates were observed visually for clear zones of haemolysis. *E. coli* BL21 carrying pET28a was used as the control.

### Sequencing of peptides resulting from *P. lautus* CtpA digestions on casein substrates

Mass spectra of peptide fragment ions, produced by CtpAp cleavage of casein substrates, were determined using the 4800 Plus MALDI TOF/TOF™ Analyzer (Applied Biosystems, CA, USA) at Proteomics Center of Fudan University (Shanghai, China). After cleavage, the digestion products were purified by the CapTrap™ Peptide column (0.5 mm i.d. × 2 mm, Michrom Bioresources, CA, USA) at a 20 μl/min flow rate with solvent A (5% acetonitrile, 95% H_2_O, 0.1% formic acid) for 5 min, and then separated by the Magic C_18_ AQ column (0.1 mm i.d. × 15 cm, 5 μm particles with 200 Å pore size, Michrom) with a linear gradient of 5 to 45% solvent B (90% acetonitrile, 10% H_2_O, 0.1% formic acid) with solvent A over 70 min at a 500 μl/min flow rate. The purified samples were subjected to the analysis with MALDI-TOF/TOF Analyzer according to the instructions of the manufacturer.

### Characterization of *P. lautus* CtpA enzyme

Purified CtpAp was subjected to several biochemical assays including optimum temperature and pH, and effects of metal ions, inhibitors and organic solvents on enzyme stability. The data was expressed as an average of the results from triplicate assays. The optimum temperature for enzyme activity was determined at temperature range of 10 to 80°C at pH 9.0 using β-casein as the substrate. The thermostability of CtpAp was assayed by incubating aliquots of purified CtpAp at 10 to 80°C for 3 h. The residual activities were measured at 30°C. The effect of pH on enzyme activity was examined at 30°C at various pH values between 3.0 to 11.0 [14,18]. The pH stability of the enzyme was assayed by incubating CtpAp at pH 3.0-10.0 at 4°C for 12 h. The residual enzyme activity was determined under standard condition at 30°C and pH 9.0 using the β-casein substrate.

The effects of metal ions (1-10 mM), detergents (0.1-0.5%), inhibitors (1-10 mM) and organic solvents (1-10%) on enzyme stability were examined by individually incubating aliquots of purified CtpAp with different reagents at 30°C for 1 h, and the residual enzyme activity was determined under standard condition as described above.

## Competing interests

The authors declare that they have no competing interests.

## Authors’ contributions

YL, YP and LC participated in the design of the study; YL carried out the major experiments, YL and LC analyzed the data; LC drafted the manuscript, and QS revised it for important intellectual content and improvement; YL, YP, QS and LC read and approved the final manuscript to be published.
